# Variations in Essential Oil Yield, Composition, and Antioxidant Activity of Different Plant Organs from *Blumea balsamifera* (L.) DC. at Different Growth Times

**DOI:** 10.3390/molecules21081024

**Published:** 2016-08-05

**Authors:** Yuan Yuan, Mei Huang, Yu-Xin Pang, Fu-Lai Yu, Ce Chen, Li-Wei Liu, Zhen-Xia Chen, Ying-Bo Zhang, Xiao-Lu Chen, Xuan Hu

**Affiliations:** 1 Environment and Plant Protection College, Hainan University, Haikou 570288, Hainan, China; yuanyuanhaida@126.com; 2 Tropical Crops Genetic Resources Institute, Chinese Academy of Tropical Agricultural Sciences, Danzhou 571737, Hainan, China; huangmei122@126.com (M.H.); 18889584013@126.com (Z.-X.C.); zhangyingbo1984@163.com (Y.-B.Z.); hillowchan@hotmail.com (X.-L.C.); mchuxuan@163.com (X.H.); 3 Hainan Provincial Engineering Research Center for *Blumea balsamifera*, Danzhou 571737, Hainan, China; 4 School of Traditional Chinese Medicine, Guangdong Pharmaceutical University, Guangzhou 510006, Guangdong, China; gyzyxzs13@126.com (C.C.); liuliwei_2013@126.com (L.-W.L.)

**Keywords:** *Blumea balsamifera*, essential oil yield, chemical composition, antioxidant activity

## Abstract

*Blumea balsamifera*, also named Ainaxiang, is widely used as an ancient medicinal herb in tropical and subtropical Asia. It is rich in essential oils. In this work the essential oils of *B. balsamifera* from different plant organs and in different months were extracted, and then analyzed by gas chromatography-mass spectrometry. The results showed that essential oil yield of young leaves was the highest (0.65 mL/100 g), followed by mature leaves (0.57 mL/100 g), and the oil yield was higher in October (0.47 mL/100 g) than other months. A total of 44 compounds were identified, representing 92.64%–96.71% of the oil. Eighteen common chemical components were found among the six plant organs, representing >80% of the oil constituents. *l*-borneol was the main ingredient in leaves, and its content was the highest in senescent leaves and in December. In the essential oils of young shoots and young stems, the main component was dimethoxydurene. Antioxidant activity was also determined using the 2,2-diphenyl-1-picrylhydrazyl (DPPH) and β-carotene bleaching (BCB) assays. The results indicated that the β-carotene bleaching activity was far stronger than the DPPH radical-scavenging capacity, and the young leaves and young shoots showed stronger antioxidant activity. Dimethoxydurene, β-caryophyllene, and α-caryophyllene play a positive role in good antioxidant activity, while β-eudesmol, phytol, and tetradecanal play a negative role. The antioxidant activity revealed in this study might help in developing this promising bioresource for use in the medicinal and cosmetic industries.

## 1. Introduction

*Blumea balsamifera* (L.) DC, also named as Ainaxiang or Dafeng’ai in China, is a perennial shrub which belongs to the Asteraceae family. It is widely distributed in China, Malaysia, Philippines, Thailand, and Vietnam. *B. balsamifera* is the most important member of the genus *Blumea* and is an indigenous herb of tropical and subtropical Asia. The whole plant or aboveground portion is usually used by native people to treat beriberi, eczema, lumbago, dermatitis, rheumatism, and skin injuries [1,2,3,4], because of its anti-microbial and anti-inflammatory activity [5,6], enhancement of percutaneous penetration activity [7], and wound healing activity [8]. It is the only plant source of Aipian, which is recorded in the Pharmacopoeia of the People’s Republic of China (2010 version). The essential oils from *B. balsamifera* are widely used in Chinese medicine and folk medicines such as Jinhoujian spray, etc. It was confirmed that certain concentrations of *B. balsamifera* oil may be safe for the liver [9]. It was also found that 100% *w/v* *B. balsamifera* oil applied to intact and damaged skin for 24 h showed no significant allergy or acute toxicity [8]. It is also widely used in the medical cosmetic and fragrances industry because of its unique fragrance. At present, various products with *B. balsamifera* as a raw material are providing great economic and social benefits [10,11].

In China, *B. balsamifera* is generally distributed in regions south of the Yangtzee River such as Hainan, Guizhou, Yunnan, Guangxi, and Guangdong provinces [12]. Due to its vast medicinal and industrial value, the constituents of *B. balsamifera* have been investigated widely, mainly focusing on the essential oils and flavonoids [13,14]. Previous studies have shown that the chemical composition and contents of *B. balsamifera* differ significantly among different plant organs, different seasons, and different places. Wang et al. [15] analyzed aroma components of *B. balsamifera* in autumn and winter using headspace-solid phase microextraction and gas chromatography mass spectrometry. They found that although the components were similar, there were large differences in the relative contents. Xia et al. [16] analyzed *B. balsamifera* from 14 different places using gas chromatography fingerprints and found similar results as Wang et al. [15]. Luo et al. found that the volatile oil content of leaves (1.1417 mL/kg) was significantly higher than in the shoots (0.6313 mL/kg) [17]. Although studies have provided some general information about the oil composition and oil content of *B. balsamifera*, studies on standards for harvesting plant materials to extract essential oils are lacking. People usually collect the leaves from September to December following traditional customs, but there is no scientifically based guidance for which month and which plant organ are the best for harvesting, so it is important to understand the variations in yield and composition of essential oils extracted from different plant organs at different growth times.

Since the period from September to December is the traditional harvest time, the primary objective of the present study was to examine the content and composition of essential oils from different plant organs of *B. balsamifera* at different growing months (from September to December), and to investigate the distribution of essential oils to provide a theoretical basis to determine the appropriate plant organ to harvest and best time of harvest. Furthermore, the antioxidant activity of the extracted essential oils was also evaluated to observe the impact of harvested plant organ and plant growth stage on the bioactive potential.

## 2. Results and Discussion

### 2.1. Results

#### 2.1.1. Yields of Essential Oil

The yields of essential oils from different plant organs in different months ranged from 0.13 mL/100 g to 0.80 mL/100 g in terms of dry weight (Table 1). The young leaves showed the highest yield (0.65 mL/100 g; mean value for four months), followed by mature leaves (0.57 mL/100 g; mean value for four months). No significant difference was noted between them (*p* > 0.05), but both showed significant differences with the remaining four plant organs (*p* < 0.05). The young stems showed the lowest yield (0.14 mL/100 g; mean value for four months), and showed a significant difference with the other five plant organs (*p* < 0.05). The oil yield was higher in October (0.47 mL/100 g), while the oil yield was the lowest in November (0.36 mL/100 g), but there was no significant difference between the four months (*p* < 0.05).

#### 2.1.2. Chemical Compositions in Essential Oil

**Figure 1:**
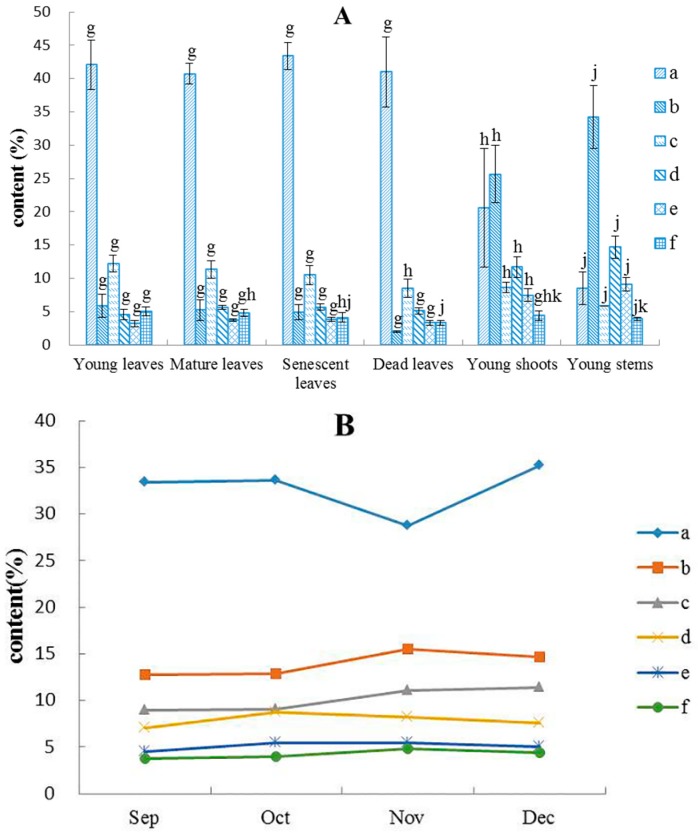
Variation in major chemical constituents in different plant organs (**A**) and months (**B**) (a: *l*-borneol; b: dimethoxydurene; c: β-caryophyllene; d: 2,2,8-trimethyltricyclo[6.2.2.01,6]dodec-5-ene; e: thujopsene-(I2); f: α-caryophyllene; g, h, j, k mean values with the same letter in the same constituent among the six plant organs are not significantly different at *p* < 0.05).

The chemical compositions of *B. balsamifera* essential oils from different plant organs and different months are presented in Table 2. Since the interaction between part of the plant and months is not significant, the average data of the plant and months was used in Table 2 and Figure 1. A total of 44 compounds were identified, representing 92.64%–96.71% of the oil. Eighteen common chemical components were found among the six plant organs, representing more than 80% of the oil. With the exception of the dead leaves, because of their low dimethoxydurene percentage, the mean concentration of the top six ingredients were: *l*-borneol, β-caryophyllene, dimethoxydurene, α-caryophyllene, 2,2,8-trimethyltricyclo[6.2.2.01,6]dodec-5-ene, and thujopsene-(I2). Figure 1 shows that the plant organs caused higher changes in the six ingredients of *B. balsamifera* essential oils than the time of growth. *l*-borneol was the main ingredient in leaves, and its content was the highest in senescent leaves (43.39%). Variations in oil yields did not show the same pattern as the percentages of *l*-borneol in the essential oil. In the essential oils of young stems and young shoots, the main composition was dimethoxydurene (34.20% and 25.64%, respectively). Except for the young stems, all the remaining five organs contained xanthoxylin, linalool, (−)-guaiol, (±)-*trans*-nerolidol, elemol, ledol, etc. The content of xanthoxylin was the highest (1.46%–3.68%). Except for the young leaves, the remaining five organs all contained dehydroaromadendrene, and the content of the young stems was the highest (1.15%). Except for the young shoots and young stems, the remaining four organs all contained 3,3-dimethyl-6-methylenecyclohexene, chrysanthenone, δ-cadinene, etc. 1-Octen-3-ol was only found in the young leaves, mature leaves, and senescent leaves. [(1*S*,2*S*,4*R*)-1,3,3-trimethyl-norbornan-2-yl] acetate and l-(+)-ascorbic acid 2,6-dihexadecanoate were only seen in the young stems, and the content of l-(+)-ascorbic acid 2,6-dihexadecanoate was higher (4.33%). (+)-α-Longipinene, (−)-globulol, linoleic acid, etc. were present only in young shoots and young stems.

The variation in main chemical compositions in different months is presented in Figure 1B. From September to December, the mean content of essential oils in different months arranged in a descending order is as follows: *l*-borneol > dimethoxydurene > β-caryophyllene > 2,2,8-trimethyltricyclo[6.2.2.01,6]dodec-5-ene > thujopsene-(I2). The content of *l*-borneol in December was the highest (35.22%) and the lowest was in November (28.73%). The content of dimethoxydurene in November was the highest (15.52%), while the content of β-caryophyllene in December was the highest (11.34%). The content of 2,2,8-trimethyltricyclo[6.2.2.01,6]dodec-5-ene was the highest in October (8.74%). There were less changes in the content of α-caryophyllene and thujopsene-(I2) during the four months.

#### 2.1.3. Antioxidant Activity

Since the interaction between part of the plant and months was not significant, the mean data of the plant and months was used in Table 3 and Table 4. From Table 3, the DPPH radical-scavenging ability of the essential oils from the six plant organs of *B. balsamifera* can be arranged in the following descending order: young leaves > mature leaves > young shoots > senescent leaves > dead leaves. The young leaves showed the strongest DPPH radical-scavenging ability (IC_50_: 3.36 ± 0.68), and a significant difference with senescent leaves and dead leaves (*p* < 0.05), while the dead leaves showed the weakest DPPH radical-scavenging ability (IC_50_: 14.86 ± 5.92), and a significant difference with the other plant organs (*p* < 0.05). The β-carotene bleaching inhibition ability of the essential oils from the six plant organs of *B. balsamifera* is arranged in descending order as follows: young shoots > young leaves > dead leaves > mature leaves > senescent leaves. The young shoots showed the strongest β-carotene bleaching inhibition ability (IC_50_: 1.81 ± 0.47), followed by young leaves (IC_50_: 1.84 ± 0.34), and they showed a significant difference with mature senescent and dead leaves (*p* < 0.05), while the senescent leaves showed the weakest β-carotene bleaching inhibition ability (IC_50_: 2.59 ± 0.68), and showed a significant difference with young and dead leaves and young shoots (*p* < 0.05). From Table 4, the DPPH radical-scavenging ability of the essential oils of *B. balsamifera* in different months can be arranged in the following descending order: December > October > November > September. The essential oils extracted in December showed the strongest DPPH radical-scavenging ability (IC_50_: 4.83 ± 2.99), and showed a significant difference with the other three months (*p* < 0.05), while the essential oils extracted in September showed the weakest DPPH radical-scavenging ability (IC_50_ value: 9.13 ± 7.31), and a significant difference with the other three months (*p* < 0.05). The β-carotene bleaching inhibition ability of the essential oils from *B. balsamifera* in different months could be arranged in descending order as follows: September > December > October > November. The essential oils extracted in September showed the strongest β-carotene bleaching inhibition ability (IC_50_: 1.98 ± 0.44), followed by December (IC_50_: 2.07 ± 0.36), and they significant differences with October and November (*p* < 0.05), while the oils extracted in November showed the weakest β-carotene bleaching inhibition ability (IC_50_: 2.43 ± 0.69), and showed a significant difference with the other three months (*p* < 0.05).

#### 2.1.4. Overall Impact of Plant Organ and Growth Time on the Essential Oils of *B. balsamifera*

**Figure 2:**
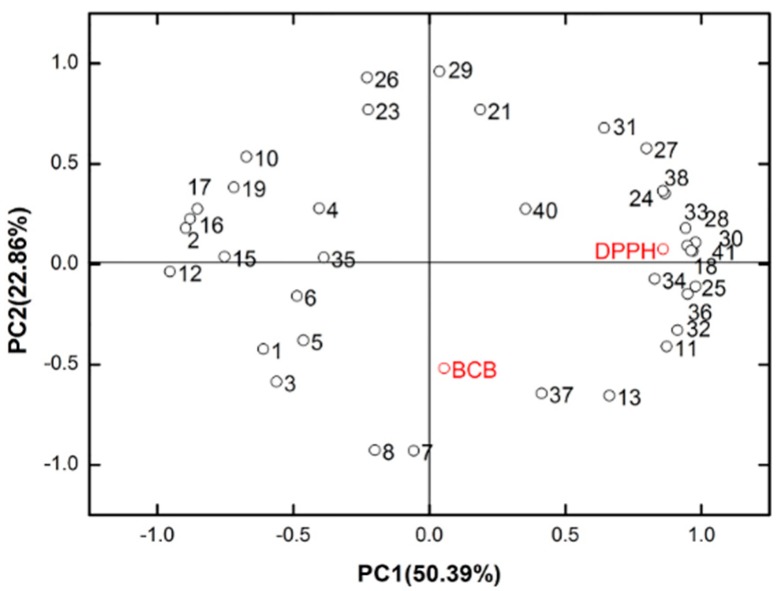
Principal component analysis of the composition and bioactivity of essential oils from the harvest *B. balsamifera* plants in different months and in different plant organs. The plane contains 73.24%. Numbers represented in the plane correspond to the compounds reported in Table 2.

The results from Figure 2 and Table 2 show that these components including dimethoxydurene (15), β-caryophyllene (16), α-caryophyllene (17), β-eudesmol (33), tetradecanal (37) and phytol (41) are related to the antioxidant activity and are relatively high in the essential oil.

Dimethoxydurene, β-caryophyllene, and α-caryophyllene play a positive role in good antioxidant activity, while β-eudesmol, phytol, and tetradecanal play a negative role.

### 2.2. Discussion

The oil yield of this plant has been researched before. Pang et al. obtained an oil yield of 3.2–4.3 mg/g (unpublished data) from plants collected in Hainan, China. He et al. reported that the oil yield is at least 2.5 mg/g in plants from Guizhou, China, and it could be increased to 7.72 mg/g if the plants were fertilized in a certain way [18]. There is no prior research discussing the differences among different plant organs at different times. The results in our research indicated that there were significant differences in oil yields of different plant organs and the same plant organ in different months. The essential oil yield of young leaves was the highest, followed by mature leaves and senescent leaves, and the oil yield was higher in October. Chen et al. found that glandular hairs are the volatile oil generating organ of *B. balsamifera*, and the density of glandular hairs is the biggest in the young leaves, and with the continuous development of leaves, the density of glandular hairs becomes smaller (unpublished conclusions).This conclusion explains well why the oil yield of young leaves was the highest.

Some previous works have studied the chemical components of the essential oils. Du et al. found that the main constituents were borneol and camphor. Others were isoborneol, terpineol, caryophyllene, eugenol, guaiol, and cubenol [19]. Chu et al. reported that 1,8-cineole was also the main constituent which accounted for 20.98% of *B. balsamifera* oil from Nanning, China [20], while in our research, we didn′t find this compound. Bhuiyan et al. found fifty constituents in the essential oils of *B. balsamifera* leaves and the main ingredients were borneol (33.22%), caryophyllene (8.24%), ledol (7.12%), and 4,4-dimethyltetracyclo[6.3.2.02,5.01,8]tridecan-9-ol (5.18%) [13].

Wang et al. [21] reported that the main constituents in essential oils of *B. balsamifera* leaves extracted by steam distillation are *l*-borneol (46.56%), xanthoxylin (8.92%), (*E*)-caryophyllene (8.75%) and (+)-γ-gurjunene (3.76%), Xia et al. found that there was big difference in the content of components of different regions by selecting *B. balsamifera* from fourteen different regions to establish a GC chromatographic fingerprint [16]. Except for *l*-borneol, the main constituents are different in the above studies. This may be because of the different origin, plant organs, different methods of extraction and GC-MS analysis conditions.

Antioxidants scavenge free radicals mainly by hydrogen atom transfer and electron transfer [22,23]. Therefore, it is more accurate to evaluate the antioxidant activity of the sample through several testing methods. In this study, the antioxidant activity was evaluated using DPPH and BCB assays, and the results proved that the essential oils of *B. balsamifera* showed a certain antioxidant activity. By comparing the IC_50_ values in the DPPH and BCB tests, it can be concluded that β-carotene bleaching inhibition activity of different plant organs and the same plant organ in different months is far stronger than the DPPH radical-scavenging capacity, which indicates that the essential oils showed stronger antioxidant activity in a lipid system. The young leaves showed stronger antioxidant activity in both the experiments, and showed a significant difference with senescent leaves and dead leaves.

To summarize the original information, for example, the impact of growth time and type of plant organ on the composition of essential oils and antioxidant activity of *B. balsamifera* plants and to identify the association between the antioxidant effects and the chemical ingredients of the essential oils from *B. balsamifera*, a PCA was used. The content of β-eudesmol and phytol was high in the essential oils extracted from dead leaves and it also had higher IC_50_ values using the DPPH method, which indicates that it possessed lower anti-radical activity. The content of tetradecanal was high in the essential oils from the senescent leaves and was also gave higher IC_50_ values using the BCB method, which indicates that it possessed lower antioxidant activity.

The content of dimethoxydurene and α-caryophyllene was high in young shoots, and the content of dimethoxydurene, β-caryophyllene, and α-caryophyllene was also high in young leaves. All of them had lower IC_50_ values in the DPPH and BCB methods, which indicate that it possessed higher antioxidant activity. These components might play a synergistic role as a good antioxidant. However, further studies should be performed to confirm the association between the antioxidant effects and the chemical ingredients of the various essential oils from *B. balsamifera*.

## 3. Materials and Methods

### 3.1. Plant Material

**Figure 3:**
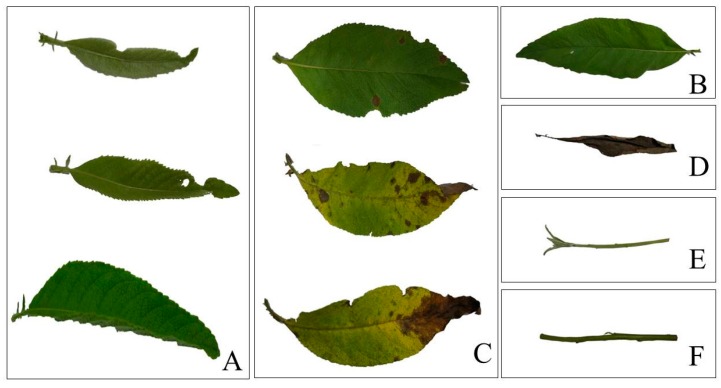
Different plant organs of *B. balsamifera* (**A**)-young leaves, (**B**)-mature leaves, (**C**)-senescent leaves, (**D**)-dead leaves, (**E**)-young shoots, (**F**)-young stems.

The experiments were performed in the experimental field of the Tropical Crops Genetic Resources Institute, Chinese Academy of Tropical Agricultural Sciences (Danzhou, Hainan, China; localization 19.52° N, 109.50° E; altitude 118 m; annual average precipitation 1815 mm; annual average temperature 23.5 °C; the soil characteristics are presented in Table 5). The experimental *B. balsamifera* plants were one-year old, and were propagated by the seeds collected from *B. balsamifera* planted in the experimental field of the Tropical Crops Genetic Resources Institute, Chinese Academy of Tropical Agricultural Sciences. They were planted with a planting spacing of 80 cm × 80 cm. On the 20th day of each month (from September 2014 to December 2014, which is the traditional harvest time), 30 one-year old *B. balsamifera* plants were randomly collected. Their young leaves (leaves on young shoots, Figure 3A), mature leaves (leaves which are mature but without yellow spots, Figure 3B), senescent leaves (leaves with yellow spots and those with dark brown leaf tips, Figure 3C), dead leaves (leaves that have turned dark brown, Figure 3D), young shoots (stems from buds to 10–20 cm part without woody parts, Figure 3E), and young stems (green stems and not completely woody, Figure 3F) were collected. These samples were divided into three parts (replicates), dried under shade, and ground to a fine powder (20-mesh sieve), packed in zip-lock bags, and stored in the refrigerator (4 °C) for oil extraction. Voucher specimens were deposited in the Herbarium of Tropical Crops Genetic Resources Institute, Chinese Academy of Tropical Agricultural Sciences.

### 3.2. Chemicals

Vitamin C and thiobarbituric acid were purchased from Solarbio (Beijing, China). 2,2-Diphenyl-1-picrylhydrazyl (DPPH) were purchased from Sigma Chemical Co. (St. Louis, MO, USA). β-carotene was purchased from Shanghai Macklin Biochemical Co. Ltd. (Shanghai, China) and butylated hydroxytoluene was purchased from Xiya Chemical Industry Co. Ltd. (Linyi. Shandong, China), All other chemicals were of analytical reagent grade.

### 3.3. Extraction of Essential Oil

The essential oils from the six plant organs in four months were extracted using hydrodistillation according to the method noted in Pharmacopoeia of the People’s Republic of China [24]. Precisely weighed *B. balsamifera* powder (200 g) was mixed with distilled water (3000 mL) The mixture was heated, and kept at a low boil for 4.5 h till the amount of oil in the vessel no longer increased, and then heating was stopped. After 1 h, the volume of essential oil was recorded. There were 24 samples and each sample was divided into three parts (replicates) after being collected from the experimental field. The percentage of essential oil yield was calculated using the formula volume of essential oil divided by the weight of sample powder. The volatile extract obtained was kept at 4 °C after drying with anhydrous sodium sulfate. The upper yellow oil was used as the sample for further analysis.

### 3.4. Analysis of Essential Oil

A GCMS-QP2010 Plus Mass Spectrometer (Shimadzu, Kyoto, Japan), equipped with a DB-5 MS capillary column (30.0 m × 0.25 mm; film thickness, 0.25 μm) and a mass spectrometry (MS) detector, was used for GC-MS analysis. The injector temperature was 250 °C. The oven temperature was programmed from 50 °C (1 min isothermal) to 180 °C at a rate of 5 °C/min and then to 250 °C at a rate of 10 °C/min, and then kept for 6 min. The interface was kept at 280 °C. The mass spectra were obtained at 70 eV. The sector mass analyzer was set to scan from 30 to 550 amu. Helium was used as a carrier gas with a flow rate of 1 mL/min. Essential oil (0.1 mL) of sample was injected (in split mode 20:1). Volatile oil components were calculated as a relative percentage of the total oil using peak area. Retention index of all the components were determined by Kovats method using *n*-alkanes (C6–C32) as standards. Identification of individual constituents was accomplished by comparing their MS spectra by matching the mass spectral data with those from the NIST (NIST 08, NIST 08s; National Institute of Standards and Technology, Gaithersburg, MD, USA), and by comparison of their MS spectra and GC retention indices with those of standard compounds available in the laboratory (β-caryophyllene, *l*-borneol and camphor) and also by comparison with some other relevant references [1,9,13,15].

### 3.5. Evaluation of Antioxidant Activity

#### 3.5.1. DPPH Radical-Scavenging Assay

First, the essential oils (separately obtained from the five plant organs in four months) and vitamin C, which was a positive control, were formulated into 1, 5, 10, 20, and 40 mg/mL ethanol solutions. The DPPH was formulated as a 1 × 10^−4^ mol/L ethanol concentration. The test solution (100 μL) and DPPH solution (200 μL) were placed in a 96-well plate as the test group. The control group contained ethanol (100 μL) and DPPH solution (200 μL), and the blank group contained test solution (100 μL) and ethanol (200 μL). After spotting was completed, the 96-well plate was placed into the Multiscan Spectrum (Multiskan GO 1.00.40, Thermo Fisher Scientific Inc., Waltham, MA, USA), shaken for 30 min, following which its absorbance was measured at 517 nm. The radical-scavenging activities (expressed as percentage inhibition of DPPH) were calculated based on the formula: inhibition percentage (Ip) = [1 − (As − Ab)/Ac] × 100%, where As, Ab, and Ac are the absorbance values of the test sample, blank sample, and control sample, respectively. The assay was repeated three times for each sample. The antioxidant activity of the essential oils was expressed in terms of half maximal inhibitory concentration (IC_50_).

#### 3.5.2. β-Carotene Bleaching Test

Antioxidant activity of the essential oils was determined on the basis of slightly modified version of the β-carotene bleaching (BCB) method [25]. β-Carotene (0.1 mg) was dissolved in chloroform (10 mL), followed by the addition of 100 mg Tween 40 and 20 mg linoleic acid. After evaporation to dryness under vacuum at 50 °C using a rotary evaporator, 50 mL oxygenated distilled water was added. The mixture was emulsified for 1 min in a sonificator to form emulsion A. The essential oils which were separately obtained from the five plant organs in four months were formulated into 0.1, 0.5, 1, 2, 3, 4, and 6 mg/mL ethanol solutions as the test samples. Next 200 μL of each sample was mixed with 5 mL of emulsion A in open-capped cuvettes; 200 μL of ethanol and 5 mL of emulsion A were prepared as the control. The absorbance of all samples was measured immediately (t = 0 min) and after 120 min on a spectrophotometer (UV-2102PC/PCS, UNIC (Shanghai) Instrument Co., Ltd., Shanghai, China) at 470 nm. The cuvettes were thermostated at 50 °C between measurements. All determinations were performed in duplicate. Another emulsion (emulsion B) consisting of 20 mg linoleic acid, 100 mg Tween 40, and 50 mL oxygenated water was also prepared. Emulsion B (5 mL) with 200 μL ethanol was used to zero the spectrophotometer. All determinations were performed in duplicate. The inhibition was calculated on the basis of the formula: % inhibition = [(A_A(120)_ − A_C(120))_/(A_C(0)_ − A_C(120))_] × 100, where A_A(120)_ is the absorbance of the test sample at t = 120 min and A_C(0)_ is the absorbance of the control at t = 0 min. A_C(120)_ is the absorbance of the control at t = 120 min. The antioxidant activity of the essential oils was expressed in terms of IC_50_.

### 3.6. Statistical Analysis

The results of the yield of essential oils and antioxidant activity were expressed as mean values ± standard deviation. One-way analysis of variance and multiple comparison methods were used to measure significant difference in the oil yield. Double factor variance analysis was used to measure significant difference in the antioxidant activity. Principal component analysis (PCA) was used to find the association between the antioxidant effects and the chemical ingredients of the essential oil which were separately obtained from the five plant organs in four months. Statistical analysis was performed using SPSS version 19.0 software (IBM Company, Chicago, IL, USA). Results were considered statistically significant when *p* < 0.05.

## 4. Conclusions

The results of this study indicate that time of growth and type of *B. balsamifera* plant organs influence the production of oil, its composition, and antioxidant activity. The essential oil level in the young leaves was the highest, followed by mature leaves and senescent leaves, and the oil content was higher in October. A total of 44 compounds were identified. In the essential oils of leaves, the main ingredient is *l*-borneol, and the content was the highest in senescent leaves and in December. Variations in oil yields did not show the same pattern as the percentages of *l*-borneol in the essential oil. In the essential oils of young shoots and young stems, the main composition was dimethoxydurene. Therefore, the time of harvest and type of plant organs should be distinguished based on the different harvesting purposes. To extract the volatile oil, the aboveground parts except stems in October should be chosen for harvest. To get a high content of *l*-borneol in volatile oil, it is more appropriate to select the leaves in December. The antioxidant activity was evaluated using DPPH and BCB assays in this study, and the results proved that the essential oils of *B. balsamifera* showed a certain antioxidant activity, and the β-carotene bleaching activity is far stronger than the DPPH radical-scavenging capacity. The young leaves and young shoots showed stronger antioxidant activity due to the high content of dimethoxydurene, β-caryophyllene, and α-caryophyllene. These results indicate that the essential oils of *B. balsamifera* have the potential to be developed into natural antioxidants for the food and cosmetics industries, while the application range and effect remain to be confirmed by further studies.

## Figures and Tables

**Table 1:** Yields (mL/100 g) of essential oils from different plant organs of *B. balsamifera* at different growth stages.

Sample	Oil Amount (mL/100 g, Dry Basis)				
	September	October	November	December	Total Mean
Young leaves	0.75 ± 0.05 ^a/fg^	0.80 ± 0.10 ^a/f^	0.61 ± 0.05 ^a/g^	0.43 ± 0.03 ^a/h^	0.65 ± 0.16 ^a^
Mature leaves	0.50 ± 0.05 ^b/f^	0.73 ± 0.07 ^a/g^	0.49 ± 0.06 ^b/f^	0.56 ± 0.05 ^b/f^	0.57 ± 0.11 ^a^
Senescent leaves	0.38 ± 0.06 ^c/f^	0.56 ± 0.12 ^b/g^	0.48 ± 0.08 ^b/fg^	0.59 ± 0.04 ^b/g^	0.50 ± 0.11 ^b^
Dead leaves	0.23 ± 0.01 ^d/f^	0.28 ± 0.08 ^cd/f^	0.23 ± 0.01 ^c/f^	0.39 ± 0.04 ^a/g^	0.28 ± 0.08 ^c^
Young shoots	0.40 ± 0.05 ^c/f^	0.30 ± 0.05 ^c/g^	0.30 ± 0.00 ^c/g^	0.21 ± 0.04 ^c/h^	0.30 ± 0.08 ^c^
Young stems	0.14 ± 0.01 ^e/f^	0.16 ± 0.01 ^d/f^	0.13 ± 0.01 ^d/f^	0.14 ± 0.01 ^d/f^	0.14 ± 0.02 ^d^
Total mean	0.40 ± 0.2 ^f^	0.47 ± 0.26 ^f^	0.36 ± 0.17 ^f^	0.39 ± 0.17 ^f^	-

^a–e^ mean values with the same letter in the same column are not significantly different at *p* < 0.05; ^f–h^ mean values with the same letter in the same row are not significantly different at *p* < 0.05.

**Table 2:** Volatile compositions of essential oils from different plant organs and different months of *B. balsamifera*.

NO.	Compound Name	RT/min		Relative Content (%)									
			RI	Young Leaves	Mature Leaves	Senescent Leaves	Dead Leaves	Young Shoots	Young Stems	Sep.	Oct.	Nov.	Dec.
1	1-Octen-3-ol	8.17	969	0.71	0.85	0.49	-	-	-	0.77	0.83	0.34	0.53
2	Linalool	11.615	1082	0.88	0.65	0.38	0.20	0.22	-	0.54	0.53	0.54	0.46
3	Chrysanthenone	12.39	1119	0.39	0.35	0.30	0.22	-	-	0.42	0.32	0.28	0.27
4	3,3-Dimethyl-6-methylene-cyclohexene	12.935	903	0.91	0.61	0.58	0.23	-	-	0.65	1.27	0.50	0.60
5	Camphor	12.98	1121	1.07	1.12	1.04	0.52	0.36	0.17	0.26	1.08	0.33	0.61
6	*l*-Borneol	13.625	1138	42.06	40.73	43.39	40.97	20.61	8.52	33.35	33.57	28.73	35.22
7	2,2,8-Trimethyltricyclo[6.2.2.01,6]dodec-5-ene	18.08	1351	4.58	5.63	5.65	5.12	11.73	14.66	7.06	8.74	8.20	7.58
8	Thujopsene-(I2)	18.59	1512	3.18	3.71	3.81	3.34	7.47	9.06	4.51	5.45	5.41	5.01
9	(+)-a-Longipinene	18.772	1403	-	-	-	-	0.26	0.38	0.25	0.28	0.36	0.38
10	Eugenol	18.845	1392	0.23	0.21	-	-	-	-	-	0.20	0.21	0.25
11	Dehydroaromadendrene	18.935	1396	-	0.11	0.16	0.24	0.35	1.15	0.54	0.47	0.36	0.33
12	Thujopsene-I3	19.425	1416	0.18	0.16	0.12	0.09	0.74	0.94	0.40	0.46	0.47	0.33
13	Dichloroacetic acid, 2-(1-adamantyl)ethyl ester	19.61	1765	0.70	0.88	0.91	0.98	2.45	3.36	1.42	1.71	1.64	1.41
14	2,3,4,5-Tetramethyltricyclo[3.2.1.02,7]oct-3-ene	19.764	1072	-	-	-	-	0.27	0.38	0.29	0.37	0.35	0.30
15	Dimethoxydurene	20.53	1511	5.85	5.23	4.95	2.01	25.64	34.20	12.78	12.86	15.52	14.68
16	β-Caryophyllene	20.62	1494	12.24	11.36	10.52	8.51	8.62	5.85	8.90	9.04	11.08	11.34
17	α-Caryophyllene	21.45	1579	5.02	4.77	4.11	3.29	4.37	3.92	3.81	3.98	4.78	4.43
18	(+)-Aromadendrene	21.635	1386	0.71	0.73	0.81	1.39	0.24	0.07	0.81	0.73	0.67	0.61
19	4-Methoxy-3-*tert*-butylphenol	22.255	1417	0.53	0.37	0.29	0.26	0.37	-	0.30	0.38	0.39	0.44
20	[(1*S*,2*S*,4*R*)-1,3,3-Trimethyl-norbornan-2-yl] acetate	23.056	1277	-	-	-	-	-	0.34	0.39	0.31	0.35	0.32
21	δ-Cadinene	23.11	1469	0.35	0.25	0.22	0.25	-	-	0.36	0.23	0.26	0.22
22	(−)-Globulol	23.132	1530	-	-	-	-	0.25	0.42	0.56	0.33	0.38	0.22
23	Elemol	23.72	1522	0.34	0.19	0.13	0.16	0.15	-	0.19	0.19	0.20	0.19
24	(±)−*trans*-Nerolidol	23.97	1564	0.48	0.38	0.38	0.61	0.25	-	0.52	0.37	0.45	0.37
25	Caryophyllene oxide	24.61	1507	1.66	2.45	3.10	5.69	1.41	2.21	3.13	2.71	2.79	2.39
26	Guaiol	24.885	1614	0.76	0.53	0.39	0.54	0.43	-	0.53	0.48	0.45	0.66
27	1-(1-Oxobutyl)-1,2-dihydropyridine	24.965	1231	0.68	0.55	0.52	0.85	0.30	-	0.65	0.50	0.61	0.55
28	Ledol	25.075	1530	0.22	0.24	0.31	0.52	0.12	-	0.36	0.26	0.28	0.25
29	γ-Maaliene	25.455	1626	0.60	0.41	0.32	0.51	0.38	-	0.49	0.40	0.37	0.52
30	α-Cuparenol	25.501	1776	-	-	0.29	0.66	0.46	2.09	1.95	0.93	0.70	0.48
31	g-Eudesmol	25.695	1626	2.34	1.84	1.79	2.41	1.70	0.40	1.92	1.77	1.96	1.80
32	Alloaromadendrene oxide-(1)	25.815	1462	0.85	1.61	2.08	2.87	0.44	0.71	1.43	1.40	1.58	1.30
33	β-Eudesmol	26.14	1593	1.63	1.66	1.66	2.91	0.92	0.27	1.79	1.48	1.49	1.28
34	Selina-6-en-4-ol	26.225	1593	1.59	1.52	1.65	2.65	-	0.43	1.89	1.46	1.52	1.25
35	Xanthoxylin	26.515	1628	3.60	3.68	3.40	1.75	1.46	-	3.91	2.92	2.46	1.90
36	3-Ethyl-3-hydroxy-androstan- 17-one	26.565	2251	0.16	0.27	0.36	0.65	0.13	0.27	0.44	0.33	0.34	0.25
37	Tetradecanal	27.34	1601	0.45	0.85	1.34	1.07	0.65	2.08	0.72	0.99	1.15	1.43
38	(−)-Spathulenol	27.63	1536	0.09	-	-	0.14	-	-	0.14	0.17	0.16	0.09
39	l-(+)-Ascorbic acid 2,6-dihexadecanoate	31.259	4765	-	-	-	-	-	4.33	3.28	4.52	5.02	4.51
40	1,7,7-Trimethylbicyclo[2.2.1]heptan-2-yl-3-methylenecyclopentane-carboxylate	31.855	1785	1.95	1.89	1.59	2.10	0.78	0.61	2.12	1.59	1.23	1.28
41	Phytol	32.995	2045	0.38	0.64	0.51	1.45	0.35	0.31	0.73	0.44	0.90	0.47
42	Linoleic acid	33.185	2183	-	-	-	-	0.14	0.96	1.33	0.40	0.93	0.53
43	Tetrahydrofuran-2-carboxylic acid, (9-oxo-9*H*-fluoren-2-yl) amide	34.821	2707	-	-	-	-	1.08	1.47	0.98	1.09	1.56	1.47
44	Cyclopropanecarboxylic acid, 1-methyl-,2,6-bis(1,1-dimethyl-ethyl)-4-methylphenyl ester	34.931	2102	-	-	-	-	0.32	-	0.40	0.35	0.23	-

RI for Retention index; - for not detected; The relative content of volatile compositions of essential oils from different plant organs were the mean value for four months, and the relative content of volatile compositions of essential oils from different months were the mean value for the six plant organs.

**Table 3:** IC_50_ value for the DPPH radical-scavenging ability and β-carotene bleaching inhibition about the essential oils of the six plant organs from *B. balsamifera*.

No.	Plant Organ	IC_50_ Value	
		DPPH	BCB
1	Young leaves	3.36 ± 0.68 ^a^	1.84 ± 0.34 ^a^
2	Mature leaves	5.05 ± 1.86 ^a,b^	2.44 ± 0.57 ^b^
3	Senescent leaves	6.11 ± 2.22 ^b,d^	2.59 ± 0.68 ^b^
4	Dead leaves	14.86 ± 5.92 ^c^	2.2 ± 0.18 ^c^
5	Young shoots	5.75 ± 0.13 ^a,d^	1.81 ± 0.47 ^a^

BCB: β-carotene bleaching; DPPH: 2,2-Diphenyl-1-picrylhydrazyl; IC_50_: half maximal inhibitory concentration. The values are expressed as mean ± standard deviation. DPPH: *n* = 12; BCB: *n* = 8. ^a–d^ mean values in the same column with the same letter are not significantly different at *p* < 0.05.

**Table 4:** IC_50_ value for the DPPH radical-scavenging ability and β-carotene bleaching inhibition about the essential oils of *B. balsamifera* in different months.

No.	Month	IC_50_ Value	
		DPPH	BCB
1	September	9.13 ± 7.31 ^a^	1.98 ± 0.44 ^a^
2	October	7.22 ± 6.04 ^b^	2.22 ± 0.63 ^b^
3	November	7.46 ± 2.53 ^b^	2.43 ± 0.69 ^c^
4	December	4.83 ± 2.99 ^c^	2.07 ± 0.36 ^a^

BCB: β-carotene bleaching; DPPH: 2,2-Diphenyl-1-picrylhydrazyl; IC_50_: half maximal inhibitory concentration. The values are expressed as mean ± standard deviation. DPPH: *n* = 15 in No. 1; *n* = 12 in No. 2–4; BCB: *n* = 10. ^a–c^ mean the values in the same column with the same letter are not significantly different at *p* < 0.05.

**Table 5:** Soil characteristics of the experimental site used in the present study.

Soil Characteristics	Experimental Field Characteristics
Organic matter (g/kg)	11.37
pH	4.94
N (g/kg)	0.51
P (mg/kg)	25.33
K (mg/kg)	33.89
